# The molecular mechanisms associated with the physiological responses to inflammation and oxidative stress in cardiovascular diseases

**DOI:** 10.1007/s12551-020-00742-0

**Published:** 2020-07-21

**Authors:** Saltanat Zhazykbayeva, Steffen Pabel, Andreas Mügge, Samuel Sossalla, Nazha Hamdani

**Affiliations:** 1 Department of Molecular and Experimental Cardiology, Ruhr University Bochum, MABF 01/597, 44780 Bochum, Germany; 2 Department of Cardiology, St. Josef-Hospital, Ruhr University Bochum, Bochum, Germany; 3 Institute of Physiology, Ruhr University Bochum, Bochum, Germany; 4 Department of Internal Medicine II, University Medical Center Regensburg, Regensburg, Germany; 5 Clinic for Cardiology & Pneumology, Georg-August University Goettingen, and DZHK (German Centre for Cardiovascular Research), partner site Goettingen, Germany; 6 Department of Clinical Pharmacology, Ruhr University Bochum, Bochum, Germany

**Keywords:** Molecular mechanisms, Cardiovascular diseases, Heart failure, Signaling pathways, Proteins modification

## Abstract

The complex physiological signal transduction networks that respond to the dual challenges of inflammatory and oxidative stress are major factors that promote the development of cardiovascular pathologies. These signaling networks contribute to the development of age-related diseases, suggesting crosstalk between the development of aging and cardiovascular disease. Inhibition and/or attenuation of these signaling networks also delays the onset of disease. Therefore, a concept of targeting the signaling networks that are involved in inflammation and oxidative stress may represent a novel treatment paradigm for many types of heart disease. In this review, we discuss the molecular mechanisms associated with the physiological responses to inflammation and oxidative stress especially in heart failure with preserved ejection fraction and emphasize the nature of the crosstalk of these signaling processes as well as possible therapeutic implications for cardiovascular medicine.

## Heart failure

**Fig. 1:**
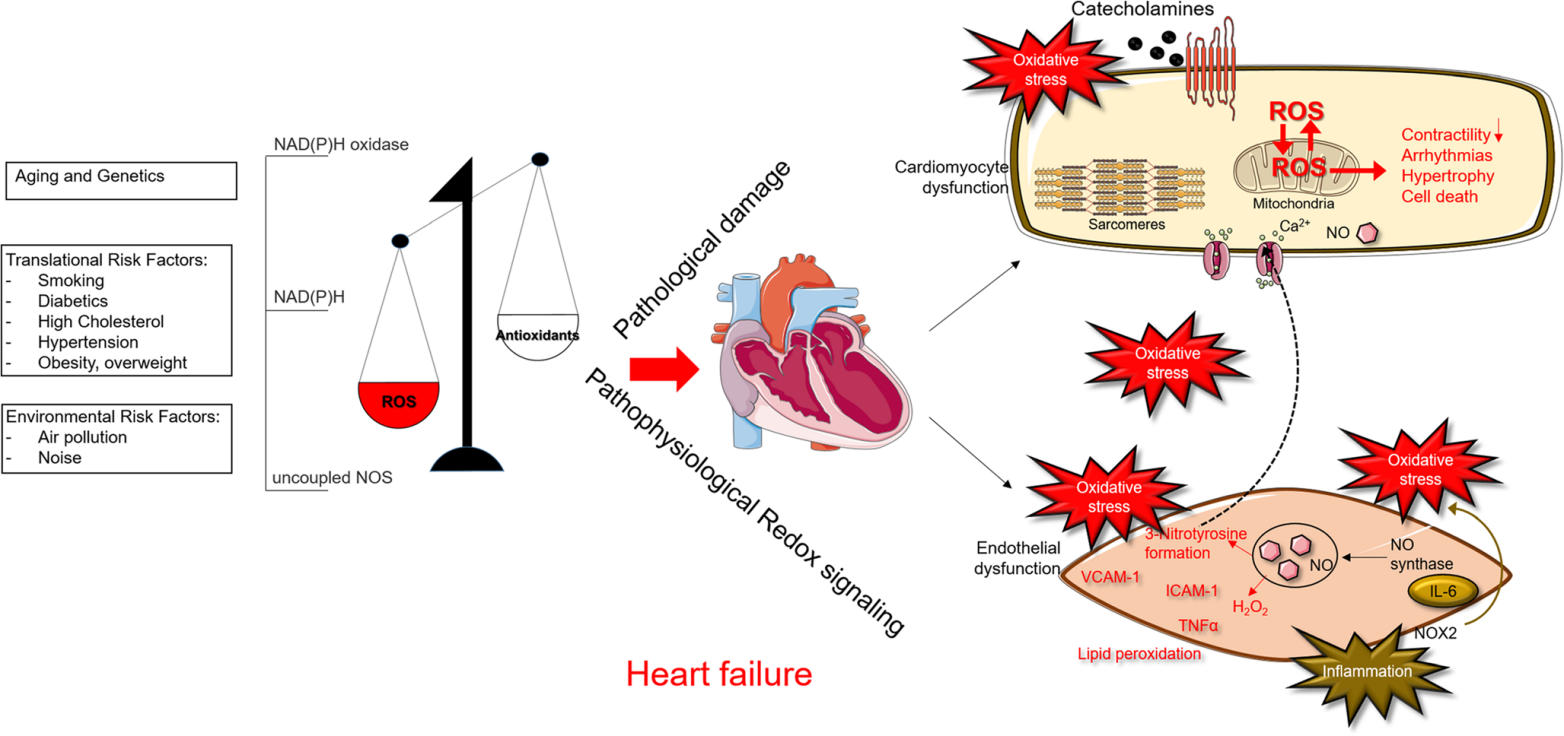
Mechanisms, sources, and implications of oxidative stress in cardiovascular disease and heart failure. Aging, genetic predisposition, conventional risk factors, and environmental factors can induce oxidative stress, where NADPH, NOX, and uncoupled NOS are dominant sources of ROS. When the generation of ROS is greater than the antioxidative capacity, then cell damage and endothelial dysfunction arise due to increased ROS level. As a consequence, oxidation of mitochondrial NADPH, H2O2 is increased, which plays a causal role in contractile dysfunction, arrhythmia, and ultimately maladaptive cardiac remodeling through hypertrophy and cell death. *Abbreviations*: H2O2, hydrogen peroxide; NADPH, nicotinamide adenine dinucleotide phosphate; NO, nitric oxide; NOS, nitric oxide synthase; NOX, nicotinamide adenine dinucleotide phosphate oxidase (NADPH oxidase); ROS, reactive oxygen species; IL, interleukin; ICAM, intercellular adhesion molecule; VCAM, vascular cell adhesion molecule; TNF-α, tumor necrosis factor-α

Heart failure (HF) is increasing in prevalence and now affects over 10 % of those aged 70 years and over. HF is characterized by the activation of the sympathetic nervous and renin-angiotensin-aldosterone systems, in a process of neuroendocrine activation that is associated with oxidative stress in the myocardium and vasculature. Oxidative stress occurs in the myocardium (Franssen et al. 2016; Maack et al. 2003; Mollnau et al. 2005) and plasma and correlates with left ventricular (LV) dysfunction (Belch et al. 1991). Inflammation and oxidative stress are well known to promote HF phenotypes (Fig. 1). The processes of inflammation and oxidative stress are through physiological interactions to the activation of downstream networks that in turn promote various human pathologies, including aging, carcinogenesis, neurodegenerative disorders, and HF associated with various causes and phenotypes (Alegre-Cebollada et al. 2014; Choudhary and Dudley Jr. 2002; Grützner et al. 2009; Kötter et al. 2014; Matough et al. 2012). To understand the role of oxidation in the pathology of disease, in particular in those diseases that show abnormalities of diastolic function, it is crucial to elucidate the functional changes that occur during oxidative stress and how they result in HF (Fig. 1).

## Heart failure with preserved ejection fraction

Heart failure with preserved ejection fraction (HFpEF) is characterized by high myocardial diastolic stiffness. Diastolic dysfunction is defined as the inability to fill the ventricle to an adequate preload volume (end-diastolic volume; EDV) at acceptably low pressures. Patients with abnormal diastolic function in particular HF with preserved ejection fraction (HFpEF) showed a complex remodeling of cardiomyocyte structure and function, in addition to a remodeling of the non-myocyte compartment (Borlaug and Kass 2006; Borlaug and Paulus 2011). Patients with abnormal diastolic function have a characteristic set of features including LV hypertrophy, concentric remodeling, increased extracellular matrix (ECM), abnormal calcium handling, abnormal relaxation and filling, and decreased diastolic distensibility. Diastolic function is often conceptualized as the totality of an active process of pressure decay (relaxation) during early diastole, which is related to myofilament dissociation and calcium reuptake, and to a “passive” stiffness dependent on viscoelastic properties, modulated by mechanical changes via the sarcomere, ECM, chamber, or pericardium (Linke and Hamdani 2014). Recent evidence suggests that oxidative stress may be the mechanistic link between obesity, diabetes mellitus, and related complications (Franssen et al. 2016; Herwig et al. 2020; Kolijn et al. 2020a; b). In obese patients, antioxidant defenses are lower than normal-weight counterparts and their levels inversely correlate with central adiposity; obesity is also characterized by enhanced levels of reactive oxygen or nitrogen species.

## Inflammation in heart failure

Classic stimuli of ventricular remodeling such as wall stress, inflammatory cytokines, and neurohormones (e.g., catecholamines and angiotensin II) induce cellular changes that are at least partially mediated via oxidative or nitrosative stress (Arstall et al. 1999; Cheng et al. 1999; Communal et al. 1998; Nakamura et al. 1998; Xiao et al. 2002). Inflammation plays a central role in the development of HF, particularly in HF with preserved ejection fraction (HFpEF). The current rise in the prevalence of HF (Ponikowski et al. 2016b) can be explained by the increasing incidence of a range of comorbidities including renal failure, arterial hypertension, chronic obstructive pulmonary disease, diabetes mellitus, and metabolic syndrome (Fig. 1). These comorbidities are usually characterized by chronic inflammation and are of particular importance for patients with HFpEF (Ponikowski et al. 2016a). Inflammation is not only critical for the development and progression of HFpEF, but the inflammatory response also plays an important role in adverse remodeling processes following myocardial infarction. The development of HF may also be directly immune-modulated, for example following autoimmune or infectious triggers such as viral infection. The inflammatory response is required to induce a regenerative response following acute myocardial injury and therefore also plays a positive role. However, sustained and chronic inflammation quickly becomes detrimental. The significance of inflammation in the development of HFpEF was firmly established in a swine model following induction of the three most common inflammation-associated comorbidities in HFpEF patients: arterial hypertension, diabetes mellitus, and hypercholesterolemia (Sorop et al. 2018). Together, these lead to diastolic dysfunction and HF, independent of coexisting coronary artery disease and hypertension. Increased inflammation is an important mechanism contributing to increased risk of HF in diabetic patients (Riehle and Abel 2016; Riehle and Bauersachs 2018). The underlying mechanisms of inflammatory-dependent HF in diabetic patients include increased expression levels of interleukins (IL) 1β and 6, intercellular adhesion molecule-1 (ICAM-1), and vascular cell adhesion molecule-1 (VCAM-1), together with decreased activity of collagen-degrading matrix metalloproteinase (Swinnen et al. 2009). Macrophages are also important mediators of inflammation and tissue remodeling in diabetes, and diverse inflammatory markers are associated with the development, diagnosis, and prognosis of patients with HF and HFpEF (Collier et al. 2011; Kalogeropoulos et al. 2010). The role of inflammation in the pathogenesis and progression of HF has important therapeutic and diagnostic implications (Briasoulis et al. 2016; Dick and Epelman 2016; Mehta and Pothineni 2016), but the question of whether inflammation is a direct cause of HF or only a marker of disease is still not completely resolved. Nevertheless, the association between the two has been shown in many studies, and pro-inflammatory biomarkers, specifically pro-inflammatory cytokines such as TNF-α, IL-1, IL-6, and galectin-3, are known to be elevated in patients with a range of phenotypes and correlate with the prognosis and severity of disease (Dick and Epelman 2016; Franssen et al. 2016; Kolijn et al. 2020b). If inflammation is the cause of many forms of heart disease, then targeting the immune response may prove beneficial in patients with signs of inflammation. If this is not the case, it seems unlikely that a treatment targeting inflammation will prove effective in HF.

A central player of inflammation in many HF patients is obesity. Obesity is accompanied by increased visceral adipose tissue, which leads to the induction of several pro-inflammatory cytokines such as tumor necrosis factor-α (TNF α), IL-6, monocyte chemoattractant protein 1, and other chemokine ligands, all of which lead to monocyte recruitment and macrophage activation. Increased peripheral inflammation, monocytosis, and monocyte differentiation to anti-inflammatory/profibrotic M2 macrophages have been associated with HFpEF in a population with a very high prevalence of metabolic comorbidities (Glezeva et al. 2015). Perivascular adipose tissue plays a major role in mediating vascular tone and endothelial inflammation through the mutual interaction of perivascular adipocytes, immune cells, vascular endothelium, and smooth muscle cells (Meijer et al. 2011). The effects can also be mediated by reduced expression of endothelial nitric oxide (NO) synthase (eNOS) and thus decreased NO synthesis, leading to reduced vasorelaxation. In addition, as obesity-associated inflammation also induces insulin resistance, an early step in the development of diabetes mellitus, comorbidities may interact. Obesity is defined as an expansion of adipose tissue as a result of excessive nutrient intake and insufficient energetic expenditure and may result in numerous different metabolic disorders including cardiovascular diseases, type 2 diabetes, and some forms of cancer. Obesity is associated with insulin resistance, which is a central component of type 2 diabetes, leading to altered glucose and lipid metabolism in adipose tissue, liver, and skeletal muscles. Insulin resistance is characterized by the failure of insulin to trigger the correct signaling mechanisms (Taniguchi et al. 2006). The chronic low-grade, systemic and local inflammation that develops during obesity links obesity to the development of insulin resistance (Gregor and Hotamisligil 2011), which then effects a variety of different organs involved in the control of metabolic homeostasis, including adipose tissue, liver, endocrine pancreas, hypothalamus, and possibly skeletal muscle. As adipocytes are a known source of pro-inflammatory cytokines, including TNF-α, IL-1, and IL-6, obesity can be seen as a chronic inflammatory condition (de Almeida et al. 2020; Franssen et al. 2016; Schmidt-Lucke et al. 2015). These cytokines are known potent stimulators of the production of ROS and NOS by macrophages and monocytes and thereby increasing oxidative stress. Adipose tissue also has the capacity to secrete angiotensin II, which stimulates NADPH oxidase activity, which in turn represents the major source of ROS production in adipocytes (Morrow 2003). Furthermore, oxidative stress causes mitochondrial abnormalities, which then further escalate overproduction of ROS. Damage to mitochondria is significant because they provide a substantial proportion of the energy required for cellular processes and also play a central role in programed cell death (apoptosis) (Wang and Nakayama 2010).

The pathways that trigger the cellular phenotypes of hypertrophy and apoptosis appear to involve stress-responsive protein kinases such as mitogen-activated protein kinases (MAPK), c-Jun N-terminal kinases, and p38 MAPKs in the myocardium (Sugden and Clerk 1998b), many of which are activated by reactive oxygen species (ROS).

### Mechanical stress as a trigger of immune activation in heart failure

The heart undergoes extensive structural and functional remodeling in response to injury, central to which is the hypertrophy of cardiac myocytes, which is characterized by the excessive deposition of extracellular matrix. Mechanical stress as a result of pressure and volume overload, together with shear stress, may induce cytokine expression and changes in cardiac extracellular matrix composition which in turn contribute to the pathogenesis of HF. In HF patients, the levels of a number of different cytokines such as TNF-α, IL-1, 6, 18, cardiotrophin-1, and Fas ligand, as well as several chemokines, are elevated in the myocardium and plasma (Aukrust et al. 1999; Meldrum 1998; Testa et al. 1996). Cytokines are not only responsible for autocrine and paracrine signaling within the myocardium but also for endocrine signaling throughout the body, especially affecting striated muscle mass through the induction of muscle wasting and cachexia. Transforming growth factor signaling and alterations to the composition of the extracellular matrix induce acquisition of a myofibroblast phenotype (Koitabashi et al. 2011). Fibrosis is accelerated as a result of intercellular interactions and crosstalk between activated fibroblasts and cardiomyocytes (Burchfield et al. 2013).

Mechanical stress resulting in LV overload can also lead to myocardial inflammation, which manifests as leucocyte infiltration and the myocardial release of pro-inflammatory cytokines (Falkenham et al. 2015; Kain et al. 2016). Pro-inflammatory cytokines affect LV function, exert a negative inotropic effect (Meldrum 1998), induce abnormalities in cardiac metabolism and energetics, promote myocardial remodeling (Diwan et al. 2003; Mann 2002; Valgimigli et al. 2001), and depress myocardial contractility. The latter may be due to uncoupling of β-adrenergic signaling, increases in cardiac NO, or alterations in intracellular calcium homeostasis (Finkel et al. 1992; Goldhaber et al. 1996; Gulick et al. 1989; Yokoyama et al. 1993). The result is cardiomyocyte hypertrophy (Yokoyama et al. 1997), necrosis, and apoptosis (Krown et al. 1996; Kubota et al. 1997), as well as activation of metalloproteinases, impaired expression of their inhibitors, and changes to the extracellular myocardial matrix, which together likely contribute to cardiac remodeling (Krown et al. 1996; Li et al. 2000; Pulkki 1997; Sivasubramanian et al. 2001). Resulting activation of the immune response also promotes the development of endothelial dysfunction, general body wasting, skeletal muscle apoptosis, and anorexia in HF (Sharma et al. 2000; Sivasubramanian et al. 2001; Torre-Amione et al. 1996). Inflammatory mediators may also contribute more indirectly to the progression of HF through impairment of bone marrow function with secondary anemia, inappropriate endothelial cell activation, and impairment of peripheral muscle, with secondary induction of systemic inflammation and reflex abnormalities in HF (Mann 2002).

Hypoxia and ischemia are additional potent inducers of inflammatory cytokines within the myocardium and their effects are primarily mediated through the production of ROS, although secondary activation of the transcriptional factor nuclear factor-κB also plays an important role (Li and Karin 1999; Singal et al. 1998). Finally, oxidized low-density lipoprotein cholesterol may increase cytokine expression in endothelial cells and monocytes, a mechanism that may be of particular importance in myocardial failure secondary to coronary artery disease (Janabi et al. 2000).

## Macrophages as key drivers of the innate immune response

Macrophages are key mediators of the innate immune response, which is involved in the recognition, phagocytosis, and elimination of pathogens. They exist within the body as both circulating and tissue-resident cells and have the ability to transform their function and phenotype based on environmental signals (Murray and Wynn 2011). Macrophages are classified as either M1 or M2 types. M1 macrophages (Murray et al. 2014) are usually associated with a pro-inflammatory response and are referred to as classically activated macrophages, with induction mediated by IFNγ, lipopolysaccharide, and TNF-α. When stimulated, M1 macrophages secrete high levels of pro-inflammatory cytokine interleukins (Martinez et al. 2008). By contrast, M2 macrophages exhibit an anti-inflammatory, pro-regenerative phenotype due to their capacity to secrete high levels of anti-inflammatory cytokines, including IL-10 and certain growth factors (Martinez et al. 2008). After angiotensin II infusion or transverse aortic constriction, macrophages are important mediators of hypertension, cardiac remodeling, and fibrosis, and depletion of macrophages results in reduced cardiac fibrosis and decreased LV hypertrophy (Falkenham et al. 2015; Kain et al. 2016). The pathological processes in many disease models are fueled by macrophage-derived cytokines (Heymans et al. 2013). Depletion of monocytes and macrophages in chronic HF models of mechanical stress prevents LV remodeling, fibrosis and preserves cardiac function (Dewald et al. 2005; Frantz et al. 2013; Kain et al. 2016; van Amerongen et al. 2007). Mice with macrophage-specific deletion of IL-10 show improved diastolic function. IL-10 may promote fibrosis by activating fibroblasts, increasing collagen deposition, and impairing myocardial relaxation (Hulsmans et al. 2018).

Inflammation promotes cardiac fibrosis in HFpEF mouse models (Glezeva and Baugh 2014; Tromp et al. 2018), and increased numbers of macrophages have been observed in HFpEF patients and appear to contribute to pathophysiology (Hulsmans et al. 2018). Fibrosis is thus heavily implicated in the development of LV diastolic dysfunction and, in addition to reduced ventricular compliance and comorbidities, may be one of the major pathophysiological mechanisms underlying HFpEF (Bode et al. 2019; Hamdani et al. 2013a; 2014; Trippel et al. 2018). Increased inflammation, elevated levels of endothelial adhesion molecules, and increased production and tissue release of inflammatory cytokines and chemokines are the earliest events found in cardiac stress states in HFpEF, including pressure and/or volume overload (Paulus and Tschöpe 2013). These states promote the infiltration of activated inflammatory cells, particularly monocytes, into cardiac tissue, and increased monocyte infiltration is also seen in hypertension and HFpEF. Once in place in tissue, monocytes differentiate into macrophages and promote cardiac inflammation, tissue injury, and myocardial fibrosis (Paulus and Tschöpe 2013). In coronary artery disease, resident macrophages distinct from monocyte-derived macrophages contribute to pathology (Honold and Nahrendorf 2018). Two mouse models of LV diastolic dysfunction, induced by either hypertension or advanced age, showed increased macrophage density in the LV compared with control mice, a finding associated with increased inflammatory monocytes (Hulsmans et al. 2018). In line with these findings, LV myocardial biopsies from patients with hypertension and HFpEF had higher macrophage densities compared with those from age-matched healthy controls (Hulsmans et al. 2018). The myocardial infiltration of inflammatory cells in HFpEF patients and in ZSF1-HFpEF rats is favored by adhesion molecules, an effect evidenced by the presence of NOX2-producing macrophages and by the high expression of CD68 (Franssen et al. 2016). In contrast to viral myocarditis, the myocardial presence of macrophages in HFpEF is not accompanied by evidence of cardiomyocyte cell death (van Heerebeek et al. 2006; 2008), perhaps due to the fact that macrophages activated due to obesity show a different pro-inflammatory phenotype (Kratz et al. 2014).

Importantly and as already mentioned, classically conceived macrophage activation proceeds via either an M1 phenotype, with potent pro-inflammatory properties, or via an M2 phenotype, with anti-inflammatory properties. However, when activated by obesity, a distinct macrophage phenotype is induced that is characterized by low levels of pro-inflammatory cytokines. Another study has also reported that the development of HFpEF is associated with monocytosis and monocyte differentiation into M2-like macrophages (Glezeva et al. 2015; Hulsmans et al. 2018). Furthermore, patients with HFpEF show increased levels of blood leukocytes and monocytes (Hulsmans et al. 2018). Taken together, these findings imply that the expansion of and phenotypic changes in cardiac macrophages may represent viable therapeutic targets when seeking to limit the cardiac inflammation that leads to diastolic dysfunction.

## What is oxidative stress?

Oxidative stress is defined based on a description of the origin of the type of ROS and other free radicals. Oxidative stress is an important contributor to tissue damage, and the cellular damage caused by oxidative stress elicits complex antioxidant defense mechanisms that have co-evolved to protect body tissues. As free radicals are constantly produced during normal metabolic processes in cells, antioxidants are also produced to ensure neutralization of free radicals. Under normal conditions, the body is usually able to maintain an equilibrium between free radicals and antioxidants.

Severe oxidative stress represents a threat to cell function and therefore results in the activation of repair mechanisms. On the other hand, disturbance in ROS levels appears to be involved in growth factor and other receptor-mediated cell signaling processes. ROS negatively affects the disposition of myocardial calcium, may induce arrhythmia, and can contribute to cardiac remodeling by inducing hypertrophic signaling, apoptosis, and necrosis (Burgoyne et al. 2012a; Wagner et al. 2013). ROS and nitric oxide (NO) produced from these sources are oxygen-/nitrogen-based chemical species with a high reactivity and include free radicals such as superoxide ion (O2 •-), hydroxyl radical (•OH), and peroxy radicals (ROO•), as well as non-radicals that are nevertheless able to generate free radicals, such as hydrogen peroxide (H2O2), nitroxyl, and NO (Breitkreuz and Hamdani 2015). A diminished capacity of nitric oxide synthase (NOS) to generate NO (Pitocco et al. 2010; Sena et al. 2013) is accompanied by increased oxidative stress during diabetic vascular dysfunction (Giugliano et al. 1996). The likely mechanism is via a superoxide ion that reacts with NO, resulting in the formation of peroxynitrite. Peroxynitrite then oxidizes the endothelial NOS (eNOS) cofactor tetrahydrobiopterin (BH4) to dihydrobiopterin (BH2), leading to eNOS uncoupling and thus producing superoxide ion rather than NO. This mechanism leads to a decrease in eNOS expression and activity in endothelial cells (Cosentino and Lüscher 1997; Srinivasan et al. 2004).

**Fig. 2:**
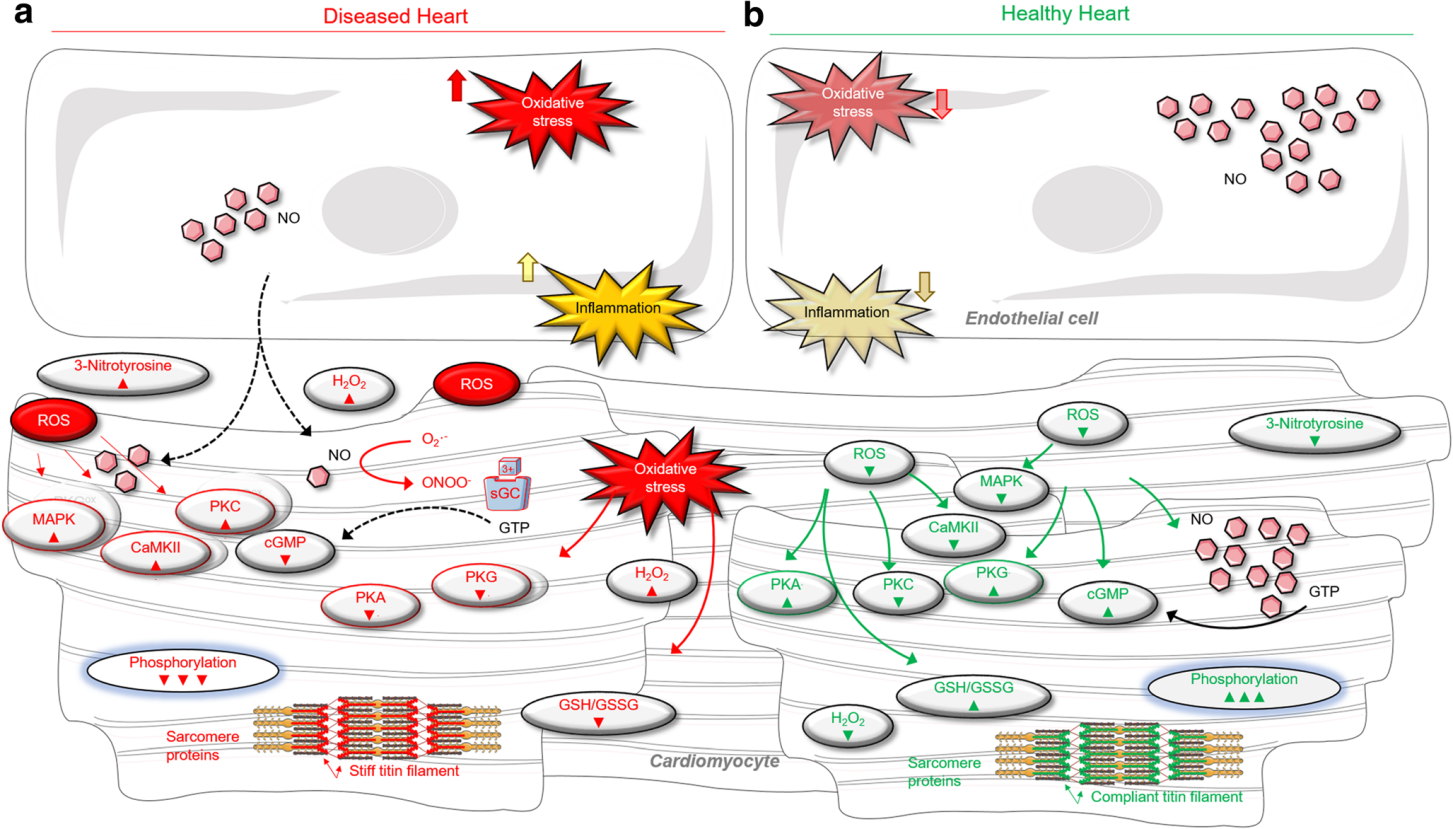
Scheme for the signaling pathways of cardiomyocyte in diseased heart under oxidative (right) and healthy (left) conditions. **A** Represents a heart under oxidative condition with impaired endothelial function via increased inflammatory cytokines and oxidative stress. **B** Represents a healthy condition showing a normal endothelial function via normal/low inflammatory cytokines and oxidative stress (green arrow pointing upwards means increase and green arrow pointing down means decrease). *Abbreviations*: cGMP, cyclic guanosine monophosphate; GTP, guanosine triphosphate; PKG, protein kinase G; H_2_O_2_, hydrogen peroxide; NO, nitric oxide; ONOO-, peroxynitrite; PKG, protein kinase G; PKA, protein kinase A; PKC, protein kinase C; CaMKII, calcium calmodulin–dependent kinase II; MAPK, mitogen-activated protein kinase; ROS, reactive oxygen species; sGC, soluble guanylyl cyclase; GSH, reduced glutathione; GSSG, oxidized glutathione

ROS are generated as metabolic by-products by biological systems (enzymatic reactions) in various cell compartments, including the cytoplasm, cell membrane, endoplasmic reticulum (ER), mitochondria, and peroxisome, all as through basal metabolic activity. In cardiomyocytes, ROS typically originate from several intracellular sources, including mitochondria, NOS, and enzymes such as xanthine oxidase, NADPH oxidase (NOX), and cytochrome p450 (Dostalek et al. 2008; Gori and Münzel 2011; Ji 2007; Srinivasan et al. 2004; Sumimoto et al. 2005; Zangar et al. 2004) (Fig. 2). Importantly, mitochondria amplify ROS derived from NOXs and may thereby function as “redox hubs” in cardiac physiology and disease (Navarro-Yepes et al. 2014; Sato et al. 2013). Additionally, many other enzymes, such as cyclooxygenases and lipoxygenases, may contribute to intracellular ROS production (Holmström and Finkel 2014). Along with resident cardiac cells, infiltrated leukocytes account for a large proportion of ROS and reactive nitrogen species (RNS) in myocardial tissues, via the production of superoxide ion and release of pro-oxidant enzyme systems like the leukocyte-derived enzyme myeloperoxidase (MPO). ROS are produced by mitochondria (Li et al. 2013; 2017; Reichart et al. 2019), which mainly takes place at the electron transport chain located on the inner mitochondrial membrane during the process of oxidative phosphorylation. Leakage of electrons at complex I and complex III from electron transport chains leads to a partial reduction of oxygen to form superoxide. Consequently, superoxide is dismutated to hydrogen peroxide by two dismutases including superoxide dismutase 2 in mitochondrial matrix and superoxide dismutase 1 in mitochondrial intermembrane space and both superoxide and hydrogen peroxide generated are considered mitochondrial ROS (Li et al. 2013).

Different signals lead to the stimulation of mitochondrial ROS such as lysophosphatidylcholine and Toll-like receptor 4 and Toll-like receptor 2 bacterial ligands lipopolysaccharide and lipopeptides; these signals are involved in regulating inflammatory response (Li et al. 2016; West et al. 2011). Among others, mitochondrial ROS adversely affects EC-coupling (Bertero and Maack 2018) and can induce cell death via activation of apoptosis and/or autophagy pathways (Finkel 2012). As previously discussed, under normal physiological conditions, the tissue oxidative balance is maintained by mobilizing an antioxidant defense system that eliminates harmful reactive oxygen/nitrogen species (ROS/NOS). Several distinct types of ROS have been identified, each of which is targeted by specific antioxidant enzymes. The one-electron reduction of O2 leads to the formation of superoxide anion, an unstable free radical that reacts with itself and other oxygen-containing species. Redox biology refers to low levels of ROS that activate signaling pathways to initiate biological processes, while oxidative stress denotes high levels of ROS that cause damage to DNA, protein, or lipids leading to different diseases.

Proteins, lipids, and DNA are the primary cellular structures affected by ROS and RNS (Wu et al. 2013). A large body of evidence shows that oxidative stress is implicated in the onset and progression of diseases such as cancer, diabetes, metabolic disorders, and atherosclerosis, in addition to cardiovascular diseases (Taniyama and Griendling 2003). Depending on the source of ROS, cell type, and tissue environment, ROS signaling may simply participate in normal physiological processes or contribute to a maladaptive response that leads to metabolic dysfunction and inflammatory signaling.

### Oxidative stress and inflammation in cardiovascular diseases

One potential trigger of HF-associated changes in muscle protein function, some of which are characterized by an increase in LV diastolic stiffness, is the oxidative stress that results from imbalances in cellular antioxidant systems and free radical production. High levels of ROS production that overwhelm cellular antioxidant defense systems are generally deleterious to contractile performance and result in adverse cardiac and skeletal remodeling. In cardiomyocytes, several intracellular sources of ROS/RNS are typically found under physiological conditions, and tissue oxidative balance is maintained by utilizing an antioxidant defense system that removes harmful ROS/NOS. The mechanism by which myocardial oxidative stress might impair cardiac function is probably oxidative damage to cellular proteins and membranes, thereby inducing cellular dysfunction or death through apoptosis and/or necrosis. However, recent studies in other organ systems indicate that ROS can exert much subtler effects, depending upon the level, the site of production, and the overall redox status of the cell (Borkowski et al. 2011; Campbell et al. 1996; Xu et al. 1998).

ROS may also activate signaling pathways that contribute to ischemic preconditioning, cardioprotection, and myocardial damage, and high levels of ROS induce structural modifications of the sarcomere that impact pump function and the general pathogenesis of HF. Many of the characteristic cellular responses found in HF can be activated by oxidative stress, including cellular hypertrophy, changes in gene expression, and cell death (Kwon et al. 2003; Siwik et al. 1999). For instance, cell growth, a hypertrophic phenotype, and apoptosis in neonatal rat cardiac myocytes in vitro can all be induced by inhibition of the antioxidant enzyme, copper-zinc superoxide dismutase (Siwik et al. 1999). Hydrogen peroxide regulates the phenotype of cardiac myocytes via concentration-dependent activation of different kinase pathways (Kwon et al. 2003), in addition to altering the turnover and properties of the extracellular matrix (Siwik et al. 2001). ROS can also exert subtler effects, depending on the level of ROS and the redox status of the cell. Regardless of the crucial role of antioxidant systems, dysregulation of oxidant signaling may cause or accelerate a host of pathological conditions. Nevertheless, the body is armed with protective measures against ROS via enzymatic superoxide dismutase, catalase, peroxiredoxin, and glutathione peroxidase, as well as non-enzymatic compounds such as vitamin E, beta-carotene, ascorbate, glutathione, and nicotinamide (Balaban et al. 2005).

Structural modifications in cardiomyocytes are caused by alterations either of protein expression, phosphorylation, or function and have been attributed to the activation of signaling pathways. In turn, these lead to changes in the magnitude of the calcium transients and an inadequate calcium-induced contractile response, or induce contractile protein modifications independent of alterations to intracellular calcium homeostasis (Adachi et al. 2004; Lancel et al. 2009; Xu et al. 1998).

### Oxidative stress and redox regulation in cardiomyocytes

Cardiac contraction is dynamically regulated on a beat-to-beat basis in order to accommodate changes in hemodynamic load and to respond to neurohumeral stresses. Control is predominantly signal-regulated via various post-translational modifications, and signaling by ROS has recently emerged as a major physiological control mechanism (Breitkreuz and Hamdani 2015). Furthermore, redox protein modifications are known to induce changes in protein structure, stability, interactivity, and activity (Fig. 2) (Disatnik et al. 1998; Gao et al. 1996; Posterino and Lamb 1996).

Myocytes isolated from the failing heart show abnormal intracellular calcium transients, along with alterations in the expression and/or activity of calcium handling proteins (Arai et al. 1993), an effect that is partly due to oxidative stress (Fig. 2). Functional changes in calcium handling are linked to post-translational modification of calcium signaling proteins, as well as oxidative and nitrosative regulation of the calcium-sensitive proteome in excitation–contraction coupling (Canton et al. 2014; Haycock et al. 1996; Posterino and Lamb 1996; Ullrich et al. 2009). ROS alter calcium transients and excitation–contraction coupling in isolated myocytes by increasing the activity of the sodium–calcium exchanger, which lead to calcium overload in human and animal models (Arai et al. 1993; Goldhaber and Qayyum 2000; Litwin and Bridge 1997). A direct effect of ROS and RNS on voltage-dependent calcium channels and calcium release channels has been noted in isolated cardiomyocytes and attributed to the activation of kinase cascades and cell death pathways, including apoptosis and necroptosis (Adachi et al. 2004; Borkowski et al. 2011; Campbell et al. 1996). ROS generation might also contribute to the activation of maladaptive signaling cascades, e.g., those leading to impaired calcium handling.

Calcium channels associated with excitation–contraction coupling regulate myoplasmic calcium levels and are organized around a system of deep membrane invaginations known as t-tubules. Depolarization of the sarcolemma leads to calcium influx into the sarcoplasm and triggers the release of calcium from the sarcoplasmic reticulum via the ryanodine receptor (calcium-induced calcium release). Changes in intracellular calcium cycling are either causal or adaptive (Bellinger et al. 2009; Durham et al. 2008; Zalk et al. 2007). Abnormalities in excitation–contraction coupling components trigger and/or aggravate contractile dysfunction and have been partially attributed to redox modifications that act as key signaling components of excitation–contraction coupling (Canton et al. 2014; Disatnik et al. 1998; Gao et al. 1996; Posterino and Lamb 1996). The redox modification of protein kinase activity and direct effects on channels and ion transporters are among the well-known effects of ROS on excitation–contraction coupling (Wagner et al. 2013).

### Modification of redox-related signaling pathways

Redox-modulated protein signaling activities that are important for cardiomyocyte function are often mediated via protein kinase A (PKA) (Brennan et al. 2006), protein kinase G (PKG) (Burgoyne et al. 2007), or calcium calmodulin–dependent kinase II (CaMKII), in addition to important contributions from the small G protein Ras (stress signaling) (Kuster et al. 2006), class II histone deacetylases (HDACs) (Ago et al. 2008), and the metabolic enzymes glyceraldehyde-3-phosphate dehydrogenase and thioredoxin (Eaton et al. 2002b; Tisdale 2002). The balance between oxidized and reduced forms of such signaling proteins is influenced by both local ROS generation and by reductants such as glutathione, which function to reduce oxidized proteins.

### cAMP-dependent protein kinase A and cGMP-dependent protein kinase G

#### Protein kinase A

β–Adrenergic activation via protein kinase A (PKA)–mediated phosphorylation targets several proteins involved in calcium handling, including the l-type calcium channel (LTCC), sarco-/endoplasmic reticulum Ca2+-ATPase (SERCA), ryanodine receptors (RyR), and myofilament proteins such as cardiac troponin I (cTnI), myosin-binding protein C (cMyBPC), and the giant protein titin (Hamdani et al. 2013a; b; 2014; 2008; 2009). Catecholamine stimulation of the β-adrenergic receptors in the myocardium plays an important role in adjusting myocardial performance to meet increased demands of the heart, as occurs upon increased stress (e.g., exercise). Phosphorylation of calcium handling and myofilament proteins regulates contractile function of the heart via its positive lusitropic effect and its contribution to an acceleration of the rate of cardiac relaxation (Fig. 2) (Gaponenko et al. 1999; Hamdani et al. 2008; Kentish et al. 2001; Lehnart et al. 2004). Alterations in PKA-mediated phosphorylation in HF have been reported following catecholamine overstimulation of the β-adrenergic receptors. In HF, the RyR2 receptors are hyperphosphorylated (Lehnart et al. 2004) and many myofilament proteins (such as cTnI, cMyBP-C, and titin) are hypophosphorylated (Hamdani et al. 2008; Solaro 2008; Solaro and de Tombe 2008) (with an exception of some phospho-sites on titin that become hyperphosphorylated when they are targeted by PKC and CaMKII, two kinases upregulated in HF) (Hamdani et al. 2013b). These changes all seem to be detrimental to cardiac performance. In addition, leakage of calcium from the SR, which increases cytosolic calcium levels during diastole and enhances myofilament calcium sensitivity, results in an increase in passive stiffness that limits relaxation of the heart muscle (Hamdani et al. 2013a; Herwig et al. 2020; Kolijn et al. 2020b; Pabel et al. 2018; van Heerebeek et al. 2008).

PKA can also be activated by redox changes acting through the formation of an inter-disulfide bond between its catalytic subunits (Brennan et al. 2006). A similar mechanism of redox activation has also been reported for PKG (Burgoyne et al. 2007). Oxidative modifications of PKA alter its activity and lead to functional and physiological consequences such as impaired insulin-stimulated lipolysis (de Piña et al. 2008). In addition, oxidation of PKA mediated by oxidants derived from NADPH oxidase 4 stimulates angiogenesis via vascular endothelial growth factor (Burgoyne et al. 2015). Modulation of cardiomyocyte stiffness via altered PKA activity has been noted in HF patients and animal models with several different types of heart disease (Hamdani et al. 2013a; Kolijn et al. 2020b). Overall, the changes noted in diseased hearts were associated with a chronic titin phosphorylation deficit related to the modulation of PKA activity. Titin can also be phosphorylated by a range of additional kinases such as protein kinase G (PKG), protein kinase C (PKC), protein kinase D (PKD), extracellular signal*–*regulated kinases (ERK), and calcium calmodulin–dependent protein kinase II (CaMKII) (Fig. 2).

#### Titin isoform switching

The titin protein consists of two main isoforms, N2BA (long and compliant) and N2B (short and stiff), and acts as a molecular spring with a crucial role in LV diastolic stiffness (Hamdani et al. 2017; Hamdani et al. 2013c; Linke and Hamdani 2014). Titin is responsible for the passive elasticity of muscle through isoform switching or post-translational modifications such as phosphorylation and oxidation. Different isoforms result from differential splicing of a single titin gene and differ in their expression level depending on the heart phenotype and disease stage. Expression of either the long and compliant N2BA or the short and stiff N2B isoform significantly influences titin-based stiffness, which then modulates diastolic function (Hamdani et al. 2017; Hamdani et al. 2013c; Linke and Hamdani 2014). By contrast, phosphorylation has rapid effects compared with isoform switching and contributes to either increased or reduced cardiomyocyte stiffness, depending on the type of kinase (Hamdani et al. 2017; Hamdani et al. 2013c; Linke and Hamdani 2014).

#### Protein kinase G

PKG regulates the function of target proteins by phosphorylating serine or threonine residues. It is a well-known modulator of diastolic function and can be modified directly and indirectly via ROS. Cyclic guanosine monophosphate (cGMP)–directed PKG signaling is initiated by soluble guanylate cyclase (sGC) or particulate GC (pGC), both lyase enzymes that convert guanosine triphosphate (GTP) to cyclic guanosine monophosphate. sGC is activated by NO, which then generates cGMP from GTP (Fig. 2) (Klaiber et al. 2011; Kuhn 2004; Moltzau et al. 2014a; b). pGC is activated by natriuretic peptides (NPs), including atrial NP and B-type NP, which bind to shared, membrane-bound GC-A or GC-C receptors. The activation of pGC can also occur through C-type NP binding to GC-B or GC-C receptors (Klaiber et al. 2011; Kuhn 2004; Moltzau et al. 2014a; b). sGC and pGC generate spatially and functionally distinct cellular pools of cGMP (Klaiber et al. 2011; Kuhn 2004) that are tightly controlled by phosphodiesterases (PDEs) (Bishu et al. 2011; Hamdani et al. 2014; Lee et al. 2015). However, the relative importance of the respective cGMP pools for HF may differ depending on comorbidities and sex-related differences, although the details of this process are still unknown. ROS can directly modify PKG via oxidation, as reduced PKGIα activity and increased PKGIα oxidation were found in human HFpEF myocardium and ZDF rats and correlated significantly with increased myocardial oxidative stress, specifically in the cytosol and mitochondria (Kolijn et al. 2020b). Studies suggested that PKGIα oxidation would likely increase the activity of the kinase (Prysyazhna et al. 2012), while other studies reported similar PKGIα activity in both WT and PKGIα Redox dead′ cysteine-42S (C42S) PKG1α knockin mice (TAC myocardium), mice harboring a knockin redox-dead mutation PKG1αC42S, perhaps due to a higher cGMP level that might blunt changes in oxidative activity (Burgoyne et al. 2007; 2012b). Furthermore, C42 oxidation reportedly reduces the capacity for activation PKGIα when countering hormone, hemodynamic (Nakamura et al. 2015), and cardiotoxic stress (Prysyazhna et al. 2016). Others have reported that the interprotomer disulfide bond is not required for oxidation-induced activation, as shown by a truncated PKGIα construct (Prysyazhna et al. 2012). Unsurprisingly, PKGIα oxidation is increased in human ischemic HF, in mouse hearts exposed to sustained pressure overload, and in canine-dilated cardiomyopathy (Nakamura et al. 2015).

**Fig. 3:**
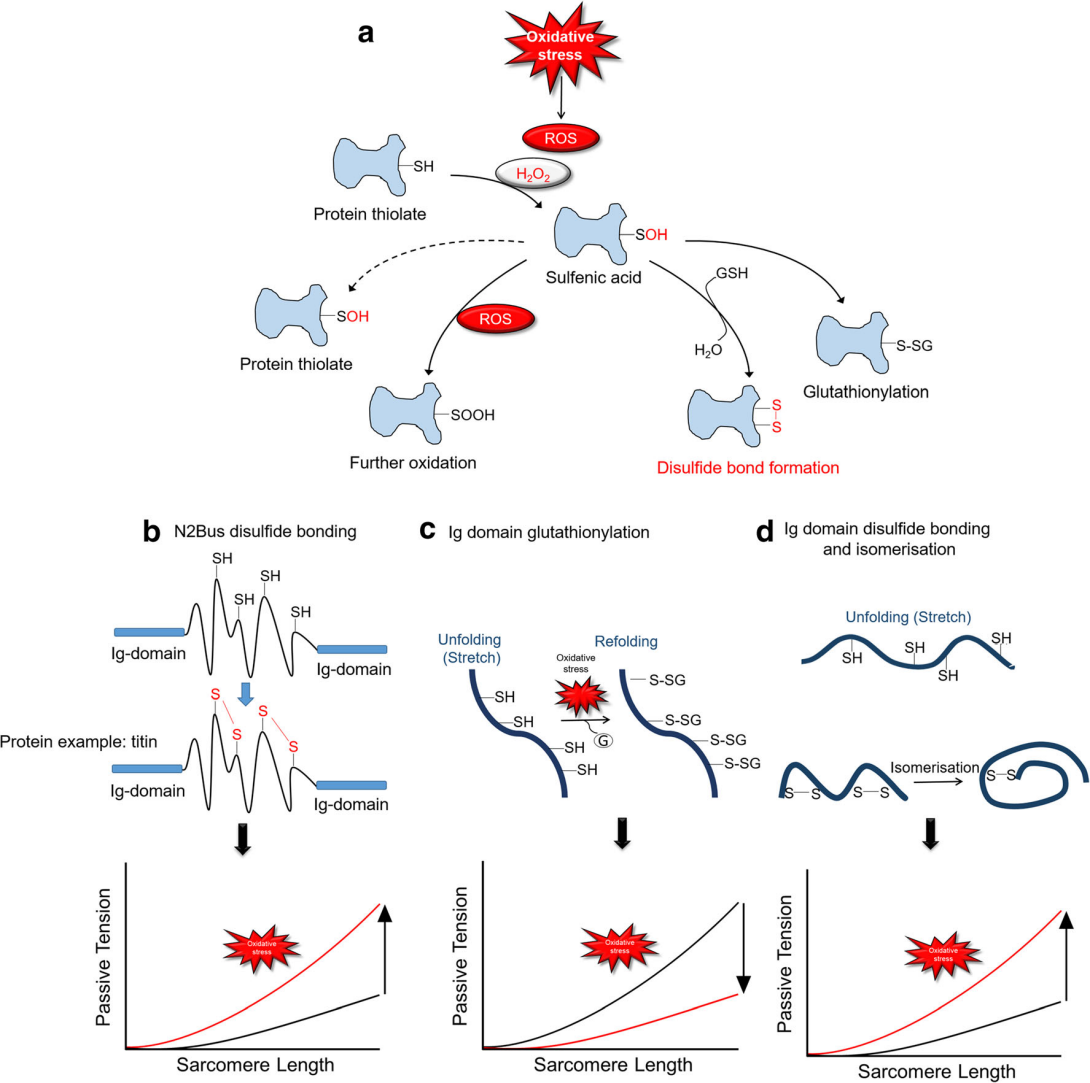
Thiol modifications of proteins and mechanisms of titin-based passive tension modulation by oxidative stress–induced titin modifications. **A** Formation of sulfenic acid from the reaction of H_2_O_2_ with protein thiolates. This formation leads to different protein modifications. In proteins without a second sulfhydryl, the sulfenic acid (–SOH) may be stabilized or will generate oxidized sulfinic (–SOOH) and sulfonic acid derivatives due to its reaction with ROS. Otherwise, a disulfide bond can form between the two sulfur atoms (–S–S–). Lastly, the sulfenated cysteinyl residue can react with glutathione (GSH), leading to a mixed disulfide. **B** Formation of intramolecular disulfide bonds within the titin-N2Bus when exposed to oxidative stress, which then increases titin-based stiffness in cardiomyocytes. **C** Ig domain unfolding due to sarcomere stretching causes exposure of hidden (“cryptic”) cysteines in Ig domains, which can become *S*-glutathionylated under oxidative conditions. This modification prevents Ig domain refolding, resulting in decreased titin-based stiffness. **D** Isomerization of disulfide bonds of the cysteine triad in titin Ig domains can occur under oxidative conditions. This modification leads to increased titin based stiffness

Modifications of cardiomyocyte function in HF are associated with alterations in cGMP-PKG signaling due to oxidative stress (indirect ROS effect on cardiomyocyte modifications) (Fig. 2). Normally, NO activates sGC in a process involving the binding of NO to the heme moiety of sGC, augmenting the action of cGMP. However, the superoxide anion is a potent inactivator of the signaling molecule NO; the resulting reduction in NO bioavailability contributes to vascular endothelial dysfunction and the loss of other physiological effects of NO (Fig. 2). The reaction between NO and superoxide generates peroxynitrite, which is itself a potent RNS. Hence, ROS can modulate the activity of various intracellular signaling pathways and molecules, potentially inducing acute and chronic modifications (Finkel 1999; Hamdani et al. 2013a; b; 2014). Signal transduction by ROS in non-phagocytic cells (Finkel 1999) can be mediated via proteins involved in myocardial excitation–contraction coupling (including ion channels, sarcoplasmic reticulum calcium–release channels, and myofilament proteins), as these proteins are prone to redox-sensitive alterations in an activity that ultimately modify cardiomyocyte function (Byrne et al. 2003; Linke and Hamdani 2014). In addition, stimulation of NO and/or activation of sGC promotes cGMP and PKG activation (Kolijn et al. 2020b; Linke and Hamdani 2014). Oxidative stress affects these pathways by lowering NO bioavailability, blocking sGC activity, increasing cGMP-specific phosphodiesterase-5A, and downregulating cGMP-PKG signaling (Franssen et al. 2016; Herwig et al. 2020; Kolijn et al. 2020b). Reduced PKG activity due to oxidative stress will then lead, among other outcomes, to hypophosphorylation of the giant titin, raising cardiomyocyte passive tension (Fig. 2). Accordingly, hearts from HFpEF patients and a small animal model of HFpEF (which exhibit reduced cGMP concentrations, reduced PKG activity, hypophosphorylated titin, and high cardiomyocyte passive stiffness) also show increased nitrotyrosine levels, indicative of nitrosative/oxidative stress (Borbély et al. 2009; Franssen et al. 2016; Hamdani et al. 2013a; b; 2014; van Heerebeek et al. 2012). These findings point to oxidative/nitrosative stress as an indirect modifier of titin phosphorylation and stiffness, which could eventually lead to diastolic dysfunction (Fig. 3). If so, many patients with HFpEF might develop diastolic dysfunction, not least because they are typically older and have various comorbidities including renal insufficiency, obesity, diabetes mellitus, or hypertension, all of which are likely to increase the level of oxidative/nitrosative stress. Taken together, these findings suggest that modulation of titin-based stiffness via cGMP-enhancing therapy could be a useful approach to correcting pathologically elevated LV diastolic stiffness, one of the primary characteristics of HFpEF in patients. Therefore, HFpEF treatment strategies might reasonably aim to treat comorbidities through the use of NO donors, phosphodiesterase-5 and 9 inhibitors, and antioxidants, the beneficial effects of which would also include a correction of titin-based myocardial stiffness.

#### Histone deacetylases

Oxidative stress is a major stimulator of epithelial cell function and can induce DNA damage and repair. During DNA repair, cells are prevented from entering S phase of the cell cycle (Clement et al. 2001). Many oxidants, including H2O2, can also induce enhanced release of inflammatory mediators from cells, a process that is associated with changes in histone acetylation (Ito et al. 2001). Histone acetylation and deacetylation are linked to cell cycle progression and correlate with repair and recombination events, as well as with gene transcription (Pazin and Kadonaga 1997; Tian et al. 2005). Histone acetylation promotes, while deacetylation inhibits, gene expression, via processes that are mediated by histone acetyltransferases and histone deacetylases (HDACs), respectively. Thioredoxin1-sensitive oxidation of the class II HDAC, HDAC4, has been implicated in α-adrenergic receptor-induced cardiomyocyte hypertrophy (Ago et al. 2008). Class II HDACs normally inhibit the transcription of prohypertrophic myocyte enhancer factor-2-dependent genes. Intramolecular disulfide formation in HDACs has been shown to regulate HDAC localization and subsequent cardiomyocyte hypertrophy (Haworth et al. 2012). Nitration of HDAC2 following oxidative stress might also account for the reduced HDAC activity found in cells from patients with oxidative stress-related diseases (Ito et al. 2001) and thus contribute to a worsening of inflammation (Ito et al. 2004).

#### Calcium calmodulin–dependent kinase II

Calcium calmodulin–dependent kinase II (CaMKII) phosphorylates several cardiac calcium handling and myofilament proteins to modulate excitation–contraction coupling, apoptosis, and gene transcription (Backs et al. 2006; Maier and Bers 2007; Toischer et al. 2010). CaMKII isoforms show increased expression/activity in failing human hearts and in animal models of cardiac hypertrophy (Anderson et al. 2011; Swaminathan et al. 2012; Toischer et al. 2010). Overexpression of CaMKIIδC in mouse myocardium is associated with massive cardiac hypertrophy and induces dilated cardiomyopathy and premature death (Zhang et al. 2003). CaMKII activity and expression are also elevated in cardiac injury models, including myocardial infarction (MI) (Singh et al. 2009; Zhang et al. 2005), and in ischemia-reperfusion (I/R) injury (Salas et al. 2010; Vila-Petroff et al. 2007). Knocking out the CaMKIIδ isoform in mice attenuates pathological cardiac hypertrophy and remodeling in response to pressure overload (Backs et al. 2009; Ling et al. 2009). CaMKII is a multimeric complex with multiple catalytic domains, therefore providing a basis for a graded calcium response (Hamdani et al. 2013c; Hudmon and Schulman 2002). Calcium-dependent activation upon oxidative stress leads to intersubunit autophosphorylation at Thr-287 within the autoinhibitory domain, preventing its reassociation with the catalytic domain and sustaining kinase activity. CaMKII activity is enhanced upon exposure to oxidative stress, as shown by the redox-active regulatory domain methionine residues (Met-281 and 282) that sustain CaMKII activity even in the absence of calcium calmodulin. Chronic angiotensin II treatment and/or myocardial infarction has been shown to promote oxidation and apoptosis (Erickson et al. 2008), but these effects were attenuated in transgenic mice expressing a CaMKII inhibitory peptide or in mice expressing Met281/282Val CaMKII. Furthermore, overexpression of CaMKII inhibitory peptide reduces CaMKII oxidation and blocks the pathological consequences of aldosterone and angiotensin II in myocardium (He et al. 2011; Purohit et al. 2013). Methionine sulfoxide reductase reduces sulfoxidized methionine residues. Investigation of mice null for methionine sulfoxide reductase A found enhanced CaMKII oxidation, cell death, and heightened sensitivity to angiotensin II and infarction (Erickson et al. 2008). This strongly suggests that oxidation of CaMKII, via ROS produced by NADPH oxidase or in mitochondria, is directly detrimental to the heart. Elimination of either of these ROS pathways, via genetic knockout or targeted ROS scavenging, results in a reduction of CaMKII oxidation (Erickson et al. 2008; Luo et al. 2013). Overexpression of CaMKII in the heart disturbs calcium homeostasis and leads to HF and arrhythmias (Maier et al. 2003; Pabel et al. 2020; Zhang et al. 2003). Taking these findings as a whole, we suggest that oxidative activation of CaMKII plays a critical role in the pathogenesis of cardiac disease.

#### Glyceraldehyde 3-phosphate dehydrogenase

Glyceraldehyde-3-phosphate dehydrogenase (GAPDH) is a conserved enzyme that controls glucose flux through the canonical Embden-Meyerhof glycolytic pathway (J Biol, 2007, vol. 6 4 pg. 10). It also mediates cell death via nuclear translocation under conditions of oxidative stress. GAPDH is a glycolytic enzyme that is responsible for the sixth step of glycolysis (Nicholls et al. 2012) and is a multifunctional protein with additional functions, including transcriptional (Zheng et al. 2003) and posttranscriptional gene regulation (Rodríguez-Pascual et al. 2008), and intracellular membrane trafficking (Chuang et al. 2005; Hara et al. 2006; Tisdale 2001). Its catalytic thiol is subject to reversible and irreversible forms of inhibitory oxidation, which have also been observed during myocardial ischemia-reperfusion (Blaustein et al. 1989; Eaton et al. 2002a). The protein undergoes S nitrosylation by NO, which triggers nuclear translocation and apoptosis (Hara et al. 2005; Sen et al. 2008). Another redox-regulated function of GAPDH is to control mRNA stabilization (Rodríguez-Pascual et al. 2008). GAPDH binds the 3′-untranslated region of endothelin-1 mRNA and enhances its degradation through destabilization. Oxidative stress alters the binding of GAPDH to this mRNA and thus its capacity to modulate endothelin-1 expression. This phenomenon occurs through specific *S*-glutathionylation of the catalytically active residue cysteine 152 (Rodríguez-Pascual et al. 2008), which also modulates the metabolic activity of GAPDH in an oxidative stress–dependent fashion (Jeong et al. 2011). GAPDH also undergoes NAD^+^ covalent linkage upon *S*-nitrosylation (Mohr et al. 1996), nitroalkylation by nitrated fatty acids (Batthyany et al. 2006), and *S*-glutathionylation by glutathione or even by NO (Mohr et al. 1999), in addition to extensive oxidation by peroxynitrite or H_2_O_2_ (Little and O'Brien 1969; Souza and Radi 1998). These results suggest that GAPDH exerts other functions beyond glycolysis and that oxidative modifications of GAPDH regulate its cellular functions by changing its interactions with other proteins.

## Oxidation of titin and its effect on cardiomyocyte stiffness

Oxidative stress, such as that seen in myocardial ischemia-reperfusion damage, obesity, or diabetes mellitus, impairs LV diastolic function, which can be independently modulated through cardiomyocyte and especially titin-based stiffness. Interestingly, the stiffness of titin can also be directly affected by oxidative stress, acting via several mechanisms.

First, oxidizing conditions promote the formation of disulfide bridges within the disordered N2-Bus element of cardiac titin. The human N2-Bus contains 6 cysteines, which can form ≤ 3 disulfide bonds. Because S–S bridges are covalent bonds, the internally cross-linked N2-Bus loses much of its extensibility, resulting in elevated cardiomyocyte passive tension (Fig. 3) (Grützner et al. 2009; Linke and Hamdani 2014).

A second direct oxidative stress–related mechanism, recently elucidated, targets the Ig domains, which constitute the majority of elastic titin. If I-band Ig domains become unfolded, for example due to increased strain on the sarcomeres, they expose cryptic cysteines which now become accessible to disulfide bonding or *S*-glutathionylation under oxidizing conditions (Fig. 3) (Alegre-Cebollada et al. 2014). Importantly, the unfolded titin-Ig domains almost exclusively form mixed disulfides with glutathione, which weakens the mechanical stability of these domains and prevents their refolding. *S*-Glutathionylation substantially reduces the passive tension of stretched human cardiomyocytes incubated with oxidized glutathione, and the effect is reversible upon incubation with reduced glutathione (Alegre-Cebollada et al. 2014). An interesting implication of this novel mechanism of regulating titin elasticity is that it opens the possibility that titin-Ig domains represent mechanosensors that respond to oxidative stress–coupled myocyte stretch with reversible mechanical softening, which could well be the origin of altered mechano-chemical signaling in stressed cardiomyocytes.

A third mechanism involves *S*-sulfenylation of cryptic cysteines, a modification that has been shown to cause titin stiffening (Fig. 3) (Beedle et al. 2016). This modification is well-known as a trigger of protein misfolding, in addition to potentially leading to the formation of a disulfide bond that protects the Ig domain fold. The intramolecular S–S bond leads to stiffening of the Ig domain segments (Ainavarapu et al. 2007; Kosuri et al. 2012), which presumably leads to increased titin-based passive stiffness in a reversible manner, depending on the redox state of the cardiomyocyte.

Finally, another mechanism of stiffness modulation under oxidative stress is the formation and isomerization of disulfide bonds in unfolded titin Ig domains. Ig domains contain many conserved cysteines which potentially oxidize under oxidative stress conditions, form S–S bridges, and isomerize (Fig. 3) (Alegre-Cebollada et al. 2011; Giganti et al. 2018; Solsona et al. 2014). This disulfide bond formation and isomerization prevents further unfolding of the Ig domain, presumably leading to increased titin-based passive tension. All of these findings point to the important role of titin oxidation in regulation of cardiomyocyte stiffness in vivo and further suggest that the correction of titin oxidation may be relevant in diseased hearts characterized by increased oxidative stress, with correction leading to reduced pathological cardiomyocyte stiffness and normalized diastolic function.

## Oxidative stress–related endothelial dysfunction in cardiovascular disease

**Fig. 4:**
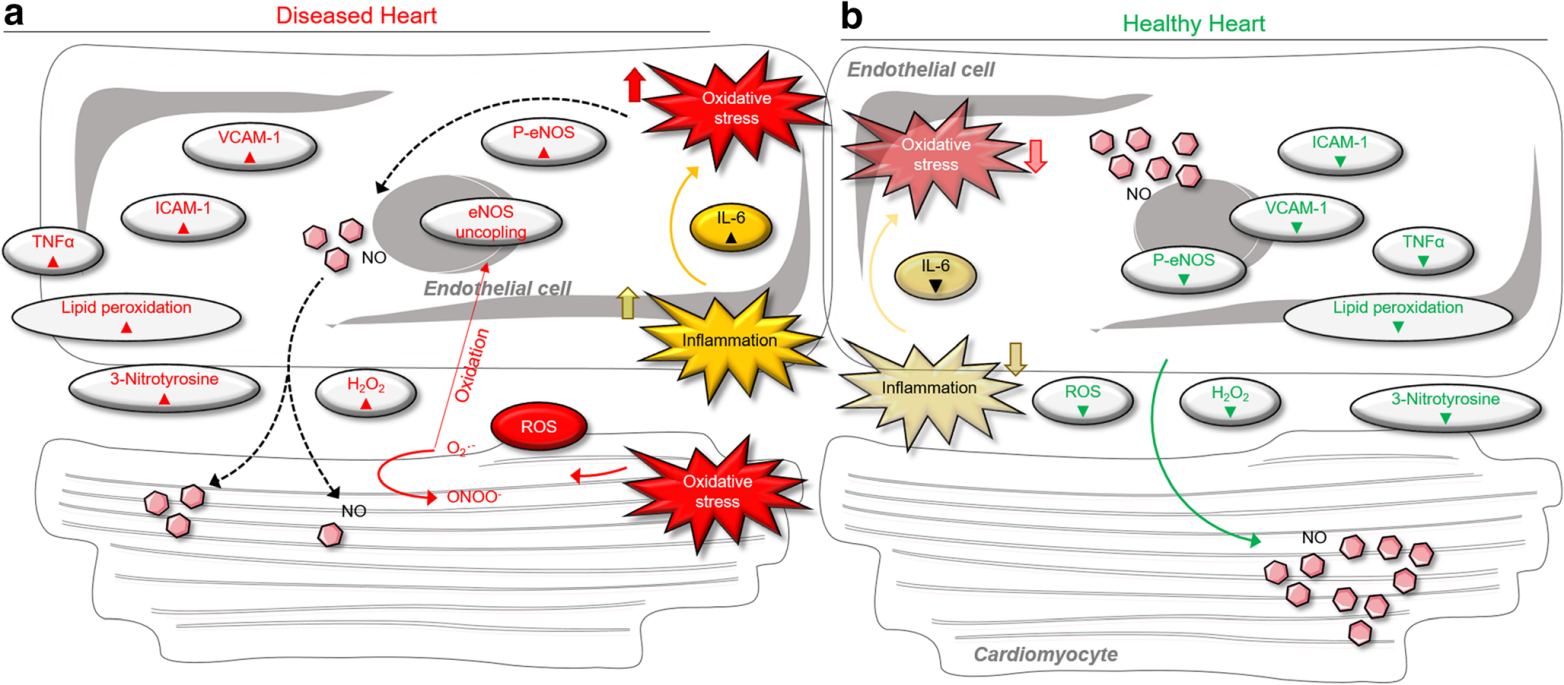
Scheme for the signaling pathways of endothelial cell in diseased heart under oxidative (right) and healthy (left) conditions. **A** Represents a heart under oxidative condition with impaired endothelial function via increased inflammatory cytokines and oxidative stress (red arrow pointing upwards means increase and red arrow pointing down means decrease). **B** Represents a healthy condition showing a normal endothelial function via normal/low inflammatory cytokines and oxidative stress (green arrow pointing upwards means increase and green arrow pointing down means decrease). *Abbreviations*: H_2_O_2_, hydrogen peroxide; ICAM-1, intercellular cell adhesion molecule-1; IL-6, interleukin-6; NO, nitric oxide; NOX2, NADPH phagocyte oxidase isoform; ONOO-, peroxynitrite; ROS, reactive oxygen species; sGC, soluble guanylyl cyclase; TNF-α, tumor necrosis factor-alpha; VCAM-1, vascular cell adhesion molecule-1; P-eNOS, phospho-endothelial nitric oxide synthase

Endothelial dysfunction is a pathological condition characterized by loss of balance in all major endothelial mechanisms (Fig. 4). The condition has been implicated in the pathophysiology of various cardiovascular diseases, including chronic HF (Endemann and Schiffrin 2004). Endothelial dysfunction is caused by inflammation, free radicals, and cytokines, acting via oxidized low-density lipoproteins that increase the expression of adhesion molecules in the endothelium, facilitating monocyte infiltration into the subendothelial space (Couillard et al. 2005; Janabi et al. 2000). Endothelial dysfunction plays an important role in the excessive systemic vasoconstriction and reduced peripheral tissue perfusion observed in chronic HF, as worsening vasoconstriction augments myocardial damage. Decreased coronary endothelium–dependent vasodilation impairs myocardial perfusion, reduces coronary flow, and worsens ventricular function. The decreased cardiac output observed in HF patients culminates in endothelial shear stress that stimulates eNOS expression. In HF, the downregulation of eNOS expression results in less NO production and hence diminished flow-mediated vasodilation, giving place to concomitant vasoconstriction. Experimental models have shown that oxidative stress significantly stimulates the progression of endothelial dysfunction (Heitzer et al. 2001). Endothelial inflammatory activation in primates developing diet-induced obesity, evident from adhesion molecule expression, appears to be the earliest manifestation of vascular damage (Chadderdon et al. 2014). Endothelial inflammatory activation is associated with microalbuminuria, which is in turn associated with diastolic dysfunction and predicts HFpEF development (Brouwers et al. 2013). In HFpEF, endothelial dysfunction is linked to the worsening of symptoms (Borlaug et al. 2010), functional capacity (Borlaug et al. 2010), and precapillary pulmonary hypertension (Farrero et al. 2014). In general, comorbidities lead to systemic inflammation and increased oxidative stress, triggering endothelial and cardiomyocyte dysfunction and so contributing to the development of HF and HFpEF (Franssen et al. 2016; Kolijn et al. 2020b). Inflammation and oxidative stress in HFpEF patients and HFpEF rats were accompanied by impaired eNOS phosphorylation and NO bioavailability in the myocardium, and specifically in cardiomyocytes (Kolijn et al. 2020b). Previous work has indicated that both are likely to be the primary underlying causes of the abnormal cardiomyocyte stiffness that results from titin hypophosphorylation (Franssen et al. 2016; Hamdani et al. 2013a; b; 2014; van Heerebeek et al. 2008). The reduced production of NO and sGC could be a result of increased H_2_O_2_ and 3-nitrotyrosine, as shown previously (Herwig et al. 2020; Kolijn et al. 2020b), and may be due to the uncoupling of eNOS, thereby switching the eNOS dimer to a superoxide anion-generating monomer (Franssen et al. 2016; Kolijn et al. 2020b).

In HF, both cardiomyocyte and endothelial cells contribute to cardiac dysfunction. Systemic inflammation induces inflammatory activation of the endothelium of myocardial microcirculation. This leads to enhanced endothelial expression of adhesion molecules such as ICAM-1, VCAM, and E-selectin (Fig. 4) (Franssen et al. 2016; Kolijn et al. 2020b). As a result of inflammatory activation, NOX2 is upregulated in endothelial cells, leading to oxidative stress, increased levels of H_2_O_2_, uncoupling of eNOS, decreased NO bioavailability, and formation of 3-nitrotyrosine and thereby endothelial dysfunction and subsequent cardiac dysfunction (Franssen et al. 2016; Kolijn et al. 2020b). On the other hand, in cardiomyocytes, increased oxidative stress leads to the generation of H2O2 and thereby decreased NO bioavailability, which in turn leads to less stimulation of sGC, reduced formation of cGMP, and diminished PKG activity (Kolijn et al. 2020b). A lack of PKG activity is associated with decreased titin phosphorylation and increased passive stiffness of cardiomyocytes. Due to a low cGMP concentration, the latter pathway fails to compensate for the decreased NO bioavailability. Ultimately, this leads to chronic cardiomyocyte dysfunction and thereby cardiac dysfunction.

## Alterations in stress signaling pathways

Biomechanical stress as a result of hypoxia, hypertension, and other forms of myocardial injury leads to a decline in myocardium function and thereby triggers signals to compensate via hypertrophy. Cardiac hypertrophy, in its early stages, is part of a compensatory response to external stressors, including mechanical loading and oxidative stresses (Linke and Hamdani 2014). The onset of cardiac hypertrophy can be a beneficial response that maintains or augments cardiac output without adverse pathology. However, when stressors persist, compensatory hypertrophy can evolve into a decompensated state with profound changes in gene expression programs, contractile dysfunction, and extracellular remodeling. A transition can occur during the dilation of the heart and thinning of the walls of the ventricular chamber.

Hyperglycemia in diabetes causes alterations in membrane and metabolic and biochemical functions that lead to contractile dysfunction and thereby cardiac dysfunction. Hyperglycemia also results in the generation of ROS, ultimately leading to increased oxidative stress in a variety of tissues. Consequently, this will lead to the activation of stress-sensitive intracellular signaling pathways once there is a redox imbalance in the cell.

One major intracellular target of hyperglycemia and oxidative stress is nuclear factor-κB (NF-κB) (Barnes and Karin 1997; Mohamed et al. 1999; Tak and Firestein 2001). NF-κB can be activated by a wide array of exogenous and endogenous stimuli including hyperglycemia, elevated free fatty acids, ROS, TNF-α, IL-1β, and other pro-inflammatory cytokines, advanced glycosylation end-product-binding and receptor for AGE, p38 MAPK, DNA damage, viral infection, and ultraviolet irradiation (Barnes and Karin 1997). In addition to its role in apoptosis, NF-κB plays a crucial role in mediating immune and inflammatory responses. Modifications in NF-κB signaling are associated with various heart diseases. The c-Jun NH(2)-terminal kinases (JNK) and p38 MAPKs are members of the extensive superfamily of MAP serine/threonine protein kinases and can be activated and/or stimulated through different stress-inducing stimuli, including oxidative stress, ROS, hyperglycemia, osmotic stress, heat shock, and pro-inflammatory cytokines (“stress-activated kinases”) (Tibbles and Woodgett 1999). These kinases are activated in response to hyperglycemia and diabetes and are involved in apoptosis, which can be suppressed by antioxidant vitamin C (Ho et al. 2000). Chronic activation of the p38 MAPK pathway is often associated with disease pathology, including inflammation, ischemia/reperfusion injury, infectious disease, and neuronal disease (Obata et al. 2000).

In addition, extracellular signal-regulated kinase (ERK) 1 and 2 cascades are markedly activated in cardiomyocytes by virtually all hypertrophic stimuli, inhibition of the cascade suppresses at least some aspects of the hypertrophic response, and constitutive activation of the pathway produces compensated cardiac hypertrophy in transgenic mice (Bueno and Molkentin 2002; Sugden and Clerk 1998a). However, while the ERK1/2 cascade is implicated in promoting cardiomyocyte hypertrophy, it can also be activated in cardiomyocytes by cellular stresses, including H_2_O_2_, which induce cardiac myocyte apoptosis. Hence, ROS, redox signaling, and oxidative stress all contribute to both physiological and pathological conditions.

## Therapeutic implications

The current treatment options for many forms of heart disease in particular for HFpEF patients are very limited and no drug has yet been shown to improve cardiac or specifically diastolic function in these patients. Many of the tested compounds were ineffective in reducing morbidity and mortality although in many cases proven to be effective for the treatment of HFrEF. Accordingly, several clinical trials have failed to show that any of these proposed drugs have positive effects in HFpEF patients. Patient management is presently limited to amelioration of symptoms and the treatment of common comorbidities such as hypertension, diabetes, obesity, and atrial fibrillation. Some trials such as the recent PARAGON-HF trial that investigated the effects of sacubitril/valsartan in patients with HFrEF showed evidence of a very heterogeneous response to treatment (Solomon et al. 2019). Potential benefit was detected only in some subgroups, such as women and patients with an ejection fraction below the median in the direction towards HF with mid-range EF. These results strongly imply that one size might not fit all in heart failure and in particular HFpEF (ClinicalTrials.gov Identifier: NCT0192071). Many studies showed suppression of several signaling pathways and in particular the cGMP-PKG pathway in HFpEF patients and animal models of HFpEF. This reduction is possibly due to inflammation and oxidative stress making it a suggestive treatment target option for these patients. However, as many heart diseases are characterized by increased inflammation and oxidative stress, one may think that targeting both could be a treatment option for many sub-groups. Indeed, our recent work suggested that increased oxidative stress and inflammation may play a major role in the deterioration of LV diastolic function, as we showed that NO-sGC-cGMP-PKG signaling is reduced in HFpEF patients and in an HFpEF animal model (Kolijn et al. 2020a; b). Increased inflammation and oxidative stress led to impaired cardiomyocyte and endothelial function and thereby to diastolic dysfunction. This latest was associated with disarranged a couple of signaling pathways and protein modifications (Kolijn et al. 2020a; b). All of which further suggest that increased oxidative stress and inflammation may exacerbate the pathophysiology of different heart diseases and specifically in HFpEF. Both acute treatments with the sodium-glucose cotransporter 2 (SGLT2) inhibitor empagliflozin and sGC activator showed antioxidant and anti-inflammatory properties when the myocardium of HF and HFpEF patients and rats were treated (Kolijn et al. 2020b). Empagliflozin is an inhibitor of the Na-dependent glucose cotransporter 2 (SGLT2) and is clinically approved as an oral antidiabetic drug. By inhibiting SGLT2 in the proximal tubule of the kidney, empagliflozin reduces the reuptake of glucose and Na and thereby lowers blood glucose. In the EMPA-REG OUTCOME trial, empagliflozin was evaluated for its cardiovascular safety. The results showed that empagliflozin reduced cardiovascular mortality, all-cause mortality, and heart failure (HF) hospitalization rates. Interestingly, the effects of empagliflozin in the EMPA-REG OUTCOME trial on HF hospitalization and mortality occurred within only a few months after initiation of treatment, suggesting the mechanisms are independent of long-acting secondary risk factors. This is supported by recent clinical data showing that empagliflozin exerts the same beneficial cardiovascular effects after adjustment for cardiovascular risk factors (blood pressure, low-density lipoprotein cholesterol, and HbA1c) (Fitchett et al. 2019; Neal et al. 2017; Wiviott et al. 2019; Zinman et al. 2015). sGC activity when the heme iron is oxidized (Fe3+) on the heme group is missing (Sandner et al. 2019; Stasch et al. 2001; 2011) and increase NO. Both empagliflozin and the sGC activator improved cardiomyocyte function via improved titin and other myofilament protein phosphorylation, an effect likely due to improved signaling pathways including the pathway NO-sGC-cGMP-PKG and the hypertrophic pathways mediated by CaMKII, PKC, and ERK2, in addition to the PKA pathway (Kolijn et al. 2020a). Moreover, treatment with both drugs reduced pathologically elevated pro-inflammatory cytokines in human HFpEF myocardium as well as in HFpEF rats and thereby improved endothelial function. This improvement was accompanied with diminished increased levels of myocardial and cardiomyocyte oxidative stress in human and rats (Kolijn et al. 2020a; b). Previous work suggested that comorbidities lead to systemic inflammation and increased oxidative stress, which triggers endothelial and cardiomyocyte dysfunction; it is not surprising that interventions that reduce inflammation are being explored as potential treatment in heart failure. Other studies also demonstrated that empagliflozin is associated with reduced inflammation and reduced activation of the nucleotide-binding domain-like 81 receptor protein 3 inflammation in liver and plasma in type 1 and 2 diabetes (Marx and McGuire 2016; Tahara et al. 2013; 2014) inflammasome in the kidney (Benetti et al. 2016) and heart (Lee et al. 2017). Previous work in HFpEF rats and human HFpEF reported diminished oxidative parameters upon empagliflozin (Kolijn et al. 2020b). Several studies showed that increased NO can suppress inflammation and regulates the synthesis of pro-inflammatory cytokines, as NO donors and l-arginine attenuate the expression of many pro-inflammatory cytokines (De Caterina et al. 1995; Spiecker et al. 1998); and supported by this the outcome of the SGLT2 inhibitors and the sGC activator in suppressing inflammation and oxidative stress, perhaps all via boosting the NO-sGC-cGMP-PKG pathway. Most importantly, these effects occur in the absence of any negative inotropy (Pabel et al. 2018). In the light of recent clinical endpoint trials investigating the effects of SGLT2-inhibitors in high-risk patients, a subgroup analysis of patients with HFpEF, which has not yet been available for these trials, would be of particular interest. However, according to the inclusion criteria of the EMPA-REG OUTCOME (Zinman et al. 2015), DECLARE-TIMI 58 (Wiviott et al. 2019), and the CANVAS study (Neal et al. 2017), a not inconsiderable number of patients should suffer from HFpEF because either the presence of established cardiovascular diseases or a combination of risk factors such as e.g. dyslipidemia or arterial hypertension in many cases combined with diabetes was inclusion criteria. The latter combination suggests a not inconsiderable proportion of HFpEF patients in the collective as a whole. Since adequate and therefore well-characterized subgroup analyses are not to be expected in this regard, the results of the DELIVER and EMPEROR-Preserved studies must be awaited, which specifically examine the influence of SGLT-2 inhibitors on hard endpoints in HFpEF patients. Finally, another potential treatment for heart failure patients with increased oxidative stress and inflammation would be theoretically “a cocktail drug,” which may combine different targets in one. As many pro-inflammatory cytokines and oxidative parameters are increased in HF, one may suggest to suppress all of them together using a multiple anti-inflammatory drug and/or a multiple antioxidant drug. In the past, the potential benefit of antioxidative strategies for treating HF has already been postulated. However, while agents with antioxidative properties i.e. like vitamin C or vitamin E showed beneficial effects in vitro, clinical trials largely failed in translating those effects into an improvement of clinical endpoints (Farías et al. 2017; Keith et al. 2001; Myung et al. 2013). A potential limitation could be that the effective concentration of the respective antioxidative agents in the heart has not been reached upon in vivo application (Farías et al. 2017). Therefore, targeting specific regulatory proteins of the pro-inflammatory and oxidative signaling cascades in cardiac disease might be a valuable approach to reduce adverse remodeling mediated by myocardial inflammation and oxidative stress. However, as individuals with HFpEF still represent a very heterogeneous collective, one may consider to establish more specific diagnostic subgroups in order to establish more precise therapeutic approaches. In this case, different combinations with the existing drugs may be more appropriate and effective for each sub-group so far.

## Conclusion

It remains very critical to understand the complex interactions of oxidative and nitrosative stress with pro-inflammatory mechanisms, metabolic dysfunction, signaling pathways, and the redox modification of proteins characteristic of heart failure to design novel approaches to therapeutic strategies for each heart failure phenotype.
